# Network Hypoactivity in ALG13-CDG: Disrupted Developmental Pathways and E/I Imbalance as Early Drivers of Neurological Features in CDG

**DOI:** 10.3390/cells15020147

**Published:** 2026-01-14

**Authors:** Rameen Shah, Rohit Budhraja, Silvia Radenkovic, Graeme Preston, Alexia Tyler King, Sahar Sabry, Charlotte Bleukx, Ibrahim Shammas, Lyndsay Young, Jisha Chandran, Seul Kee Byeon, Ronald Hrstka, Doughlas Y. Smith, Nathan P. Staff, Richard Drake, Steven A. Sloan, Akhilesh Pandey, Eva Morava, Tamas Kozicz

**Affiliations:** 1 Department of Clinical Genomics, Mayo Clinic, Rochester, MN 55905, USA; rameen.shah@stonybrookmedicine.edu (R.S.); chandran.jisha@mayo.edu (J.C.); 2 Department of Biochemistry and Molecular Biology, Mayo Clinic, Rochester, MN 55905, USA; 3 Department of Genetics and Genomic Science, Icahn School of Medicine at Mount Sinai, New York, NY 10028, USA; 4 Renaissiance School of Medicine, Stony Brook University, Stony Brook, NY 11794, USA; 5 Department of Laboratory Medicine and Pathology, Mayo Clinic, Rochester, MN 55905, USA; 6 Department of Genetics, Section Metabolic Diagnostics, University Medical Center Utrecht, 3584 EA Utrecht, The Netherlands; 7 Department of Human Genetics, Emory University, Atlanta, GA 30322, USA; 8 Biochemical Genetics Department, Human Genetics and Genome Research Institute, National Research Centre (NRC), Cairo 12622, Egypt; 9 Department of Cell and Molecular Pharmacology and Experimental Therapeutics, College of Medicine, Medical University of South Carolina, Charleston, SC 29425, USA; 10 Hollings Cancer Center, Medical University of South Carolina, Charleston, SC 29425, USA; 11 Manipal Academy of Higher Education, Manipal 576104, Karnataka, India; 12 Institute of Bioinformatics, Bangalore 560066, Karnataka, India; 13 Department of Neurology, Mayo Clinic, Rochester, MN 55905, USA; 14 Center for Regenerative Medicine, Mayo Clinic, Rochester, MN 55905, USA; 15 Department of Biophysics, University of Pécs Medical School, 7624 Pécs, Hungary; 16 Department of Anatomy, University of Pécs Medical School, 7624 Pécs, Hungary

**Keywords:** congenital disorders of glycosylation (CDG), ALG13 deficiency, N-linked glycosylation, cortical brain organoids, infantile spasms and epilepsy, excitatory–inhibitory imbalance, network hypoactivity, multi-omics analysis, X-chromosome inactivation skewing

## Abstract

Background: ALG13-CDG is an X-linked N-linked glycosylation disorder caused by pathogenic variants in the glycosyltransferase ALG13, leading to severe neurological manifestations. Despite the clear CNS involvement, the impact of ALG13 dysfunction on human brain glycosylation and neurodevelopment remains unknown. We hypothesize that ALG13-CDG causes brain-specific hypoglycosylation that disrupts neurodevelopmental pathways and contributes directly to cortical network dysfunction. Methods: We generated iPSC-derived human cortical organoids (hCOs) from individuals with ALG13-CDG to define the impact of hypoglycosylation on cortical development and function. Electrophysiological activity was assessed using MEA recordings and integrated with multiomic profiling, including scRNA-seq, proteomics, glycoproteomics, N-glycan imaging, lipidomics, and metabolomics. X-inactivation status was evaluated in both iPSCs and hCOs. Results: ALG13-CDG hCOs showed reduced glycosylation of proteins involved in ECM organization, neuronal migration, lipid metabolism, calcium homeostasis, and neuronal excitability. These pathway disruptions were supported by proteomic and scRNA-seq data and included altered intercellular communication. Trajectory analyses revealed mistimed neuronal maturation with early inhibitory and delayed excitatory development, indicating an E/I imbalance. MEA recordings demonstrated early network hypoactivity with reduced firing rates, immature burst structure, and shortened axonal projections, while transcriptomic and proteomic signatures suggested emerging hyperexcitability. Altered lipid and GlcNAc metabolism, along with skewed X-inactivation, were also observed. Conclusions: Our study reveals that ALG13-CDG is a disorder of brain-specific hypoglycosylation that disrupts key neurodevelopmental pathways and destabilizes cortical network function. Through integrated multiomic and functional analyses, we identify early network hypoactivity, mistimed neuronal maturation, and evolving E/I imbalance that progresses to compensatory hyperexcitability, providing a mechanistic basis for seizure vulnerability. These findings redefine ALG13-CDG as disorders of cortical network instability, offering a new framework for targeted therapeutic intervention.

## 1. Introduction

**Figure 1:**
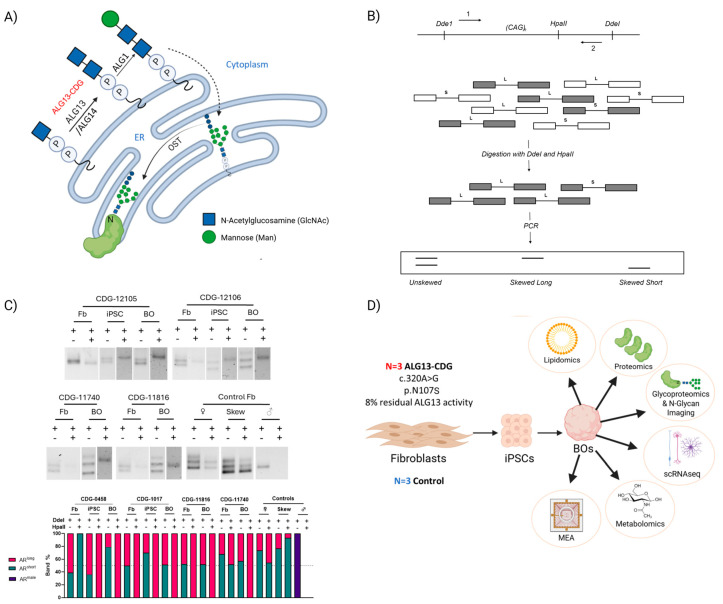
Background and Study Design (**A**) Protein N-linked glycosylation pathway in the ER, showing the role of ALG13. (**B**) Schematic summary of the X-inactivation assay. Unmethylated euchromatin (white) will be digested by HpaII and will not amplify. The undigested methylated heterochromatin (gray) will be amplified, and relative abundances of the long and short polygenic AR CAG repeat regions will indicate the presence of X-inactivation skewing. (**C**) X-inactivation skewing in ALG-13-CDG fibroblasts, induced pluripotent stem cells (iPSCs), and human cortical brain organoids (hCOs). DNA agarose gel electrophoresis of PCR amplicons of the androgen receptor (AR) exon1 CAG trinucleotide repeat region in gDNA incubated with DdeI and HpaII from fibroblasts collected from ALG13-CDG fibroblasts, iPSCs, and hCOs, as well as fibroblasts from a male control, a female control, and a female control with clinically assayed 90% skewed X-inactivation. The relative abundance in % of total band abundance of the polygenic alleles amplified by PCR in DNA incubated with DdeI and HpaII. (**D**) Workflow for study. BOs, brain organoids; MEA, Multi-Electrode Array.

Congenital Disorders of Glycosylation (CDGs) are a growing group of inherited conditions caused by defects in protein and lipid glycosylation and other glycosylation-related pathways, with more than 160 distinct disorders identified to date [1]. While CDGs often impact multiple organ systems, a substantial proportion present with predominant neurological symptoms, including developmental delay, epilepsy, and intellectual disability [2]. Asparagine-linked glycosylation 13 (ALG13) is a UDP-GlcNAc transferase that catalyzes a key step in N-linked glycosylation by adding N-acetylglucosamine (GlcNAc) to the glycan chain in the endoplasmic reticulum (Figure 1A). Variants in the glycosyltransferase domain of ALG13, particularly c.320A>G, p.N107S, lead to ALG13-CDG, a severe disorder of glycosylation with predominantly neurological manifestations, including developmental delay, intellectual disability, seizures, and central hypotonia [3,4,5,6,7,8]. ALG13-CDG is an X-linked condition, often with de novo mutations in females, while males experience early lethality, suggesting an X-linked dominant pattern [9].

Currently, management of ALG13-CDG symptoms is limited to seizure control with anti-epileptic drugs, ACTH treatment, and ketogenic diets, which often yield only partial relief [4,5,6]. Unlike other N-linked CDGs, ALG13-CDG is not detectable through standard glycosylation profiles, requiring genetic testing for diagnosis. Although studies in ALG13-deficient yeast have demonstrated glycosylation defects [9], the specific impact on brain glycosylation in humans remains unexamined. We hypothesized that ALG13-CDG may cause organ-specific glycosylation impairment, particularly within the brain, correlating with the neurological symptoms observed in affected individuals.

In this study, we reprogrammed patient-derived fibroblasts into induced pluripotent stem cells (iPSCs; Figure 1D) and generated three-dimensional human cortical organoids (hCOs) to study ALG13-CDG in a model system that reflects the human disease. Using this model, we performed high-resolution functional electrophysiology (Multi-Electrode Array) and molecular analysis, including glycoproteomics, N-glycan imaging, proteomics, scRNAseq, metabolomics, lipidomics, and, alongside assessments of X-inactivation patterns (Figure 1B–D).

Our findings reveal previously unidentified glycosylation defects, skewed X-inactivation, and altered gene expression and lipid profiles in ALG13-CDG, providing new insights into the disease mechanism and potential therapeutic targets.

By integrating functional and molecular data, we uncovered critical insights into how glycosylation defects reshape neurodevelopmental pathways and trajectories, disrupt excitatory/inhibitory balance, and impair network maturation. Although this work centers on ALG13-CDG, the pathway changes and mechanisms identified, such as mistiming of neuronal lineage maturation and cortical network hypoactivity, may represent convergent features across CDGs with prominent neurological involvement. This study thus provides a framework for linking molecular pathology to network-level dysfunction in glycosylation disorders, with potential implications for future therapeutic strategies.

## 2. Methods

### 2.1. Ethics

We collected the genetic, laboratory, and clinical data of 4 patients with ALG13-CDG (Supplementary Table S1) enrolled in the Frontier in CDG Consortium (FCDGC) natural history study (IRB: 19–005187; NCT04199000). All patients’ skin biopsies were obtained for establishing fibroblasts as part of the standard clinical care. Informed research content was obtained from all patients included in the study. Residual samples from ALG13-CDG-affected individuals (CDG-1017, CDG-0458, CDG-11740, and CDG-11816) were deidentified and used for further analysis. Additional healthy fibroblasts were obtained from the Coriell Institute (CTRL-5400, CTRL-5381, and CTRL-8399). CTRL-1363.1 and CTRL-8856 iPSCs were donated by Dr. Sergiu Pasca.

Reprogramming of ALG13-deficient fibroblasts to induced pluripotent stem cells and differentiation of ALG13-deficient cortical organoids.

The iPSCs were reprogrammed and cultured as previously described [10]. ALG13-CDG iPSCs were assessed for pluripotency markers and ability to differentiate into all 3 germ layers (Supplementary Figure S1). All our controls have also been previously validated for pluripotency [10]. iPSC cells were cultured in a 10 cm plate, and the organoids were developed using the dispase method as previously described [10].

### 2.2. X Inactivation Skewing

Genomic DNA (gDNA) from fibroblasts, iPSCs, and cortical organoids was isolated using the QIAamp DNeasy Mini Kit (Qiagen, MD, USA). X-inactivation skewing was assessed using the Cutler Allen et al. method [11] with slight modifications described in the Supplementary Methods.

X inactivation results in hypermethylation of one X chromosome, preventing transcription. An HpaII restriction site at c.109_112 in AR exon 1 (Figure 1B) allows for digestion of active, unmethylated sequences, which are not amplified by PCR. Methylated, inactivated sequences escape digestion and are amplified. By comparing the two alleles’ abundance after PCR, X-inactivation skewing can be assessed (Figure 1B).

### 2.3. Organoid Lysis and Protein Digestion

Five hCOs from each ALG13-CDG cell line (CDG-11740, CDG-11816, and CDG-1017) and from control lines (CTRL-5381, CTRL-5400, and CTRL-8856.1) at day 77 were collected and washed 3 times with DPBS, flash frozen in dry ice, and kept at −80 °C till time of assay. The samples were lysed using a Bioruptor sonication device in 8 M urea (in 100 mM TEABC) with 1% protease inhibitor cocktail (Thermo Scientific, MA, USA). Protein amount was quantified in the organoid lysates using the BCA colorimetric assay as per the manufacturer’s instructions (Thermo Scientific). An equal quantity of protein from both ALG13-CDG and controls was first reduced using 10 mM TCEP for 30 min at 55 °C on a thermomixer and then alkylated with 40 mM iodoacetamide for 30 min in the dark at room temperature. The proteins were then digested, desalted, and tandem mass tag (TMT) labeled as previously described [10].

Peptide fractionation, glycopeptide enrichment, liquid chromatography tandem mass spectrometry (LC-MS/MS), and data analysis.

Twenty percent of the peptides were fractionated using bRPLC for proteomics, and 80% of the peptides were fractionated using size-exclusion chromatography (SEC) for glycoproteomics, as previously described [10]. LC-MS/MS for glycoproteomics and proteomics was conducted as previously described [10] with slight alterations provided in the Supplementary Methods. Data analysis for glycoproteomics and proteomics was performed as described previously [10].

### 2.4. Tissue Preparation, Processing, and Analysis for MALDI-MSI

CDG-11740, CDG-11816, CDG-1017, CTRL-1363.1, CTRL-8399, and CTRL-8856.3 hCOs at day 145 were preserved as described [12]. Spheroids were sectioned at 12 um onto glass slides. Samples were prepared similarly to a standardized protocol as described [13]. Tissue samples were analyzed on a timsTOFfleX MALDI-QTOF as described in the Supplementary Methods.

### 2.5. Single Cell Suspension Formation, Library Preparation, and Sequencing for scRNAseq

At day 92, four organoids per cell line (CDG-0458, CDG-1017, CDG-11740, CTRL-8856.3, CTRL-1363.1, CTRL-8399) were dissociated into single cells using the Miltenyi Biotec Neurosphere Dissociation Kit, with a modified 20–25 min incubation at 37 °C. Cells were resuspended in 1 × PBS + 0.04% BSA and sent to the Genome Analysis Core (GAC) for single-cell partitioning. Library preparation and sequencing details are in the Supplementary Methods.

### 2.6. scRNAseq Data Analysis

Details on how fastq files were used to generate an integrated Seurat object can be found in the Supplementary Methods. After integration, the data was scaled with ScaleData, and PCA was performed. Clustering analysis using FindNeighbors, FindClusters, and UMAP (dimensions 1:20) identified eight distinct clusters. Cluster identities were determined using FindAllMarkers and FindConservedMarkers and matched with the literature [14,15,16,17]. Seven of the clusters were identified according to their markers (Figure S4). One cluster remained unclassified due to a lack of definitive markers.

After cluster identification, differential gene expression analysis was performed by aggregating data into pseudobulk samples per organoid line and running DESeq2 with the apeglm method [18] to identify significant transcript changes using padjusted values < 0.05. The integrated Seurat object was split into ALG13-CDG and control sample objects for trajectory analysis using Monocle3 [19]. Following this analysis, the previously unidentified cluster was reclassified as multipotent progenitor cells (MPC) due to its branching toward inhibitory and astrocyte progenitor cell populations.

### 2.7. CellChat Analysis

The cell–cell interactions between the different cell types in the organoid were evaluated using CellChat (R package, v2) [20]. First, the split Seurat object was used to generate CellChat objects for controls and ALG13-CDG. The CellChatDB.human, which is a database that contains information about known interactions between receptors, ligands, and cofactors, was integrated into the CellChat objects to allow the determination of cell–cell communication networks. The CellChat data was processed as previously described [20].

### 2.8. Organoid Preparation and Metabolite Measurement for Metabolomics

Five cortical organoids from each ALG13-CDG cell line (CDG-11740, CDG-11816, and CDG-1017) and from control lines (CTRL-5381, CTRL-5400, and CTRL-8856.1) were collected and washed 3 times with DPBS, flash frozen in dry ice, and kept at −80 °C till time of assay. At the time of the assay, hCOs were washed with saline and weighed. A total of 350 µL of ice-cold extraction buffer (80% MeOH, internal standards) and a few sonication beads were added to the sample and placed in the Bioruptor sonication device for lysing. After lysing, the metabolite extraction, LC-MS, and normalization were conducted as previously described [10].

### 2.9. Lipid Extraction from Organoids

Untargeted lipidomics analysis was performed on cortical organoids from three ALG13-CDG lines (CDG-11740, CDG-12105, and CDG-12106) and five control lines (CTRL-5400, CTRL-5381, CTRL-8399, CTRL-1363.1, and CTRL-8856). Deuterated lipids were added prior to conducting a modified Bligh and Dyer lipid extraction [21]. The dried organic layer was reconstituted in chloroform (1:3 *v*/*v*) for LC-MS/MS analysis.

### 2.10. LC-MS/MS Analysis of Lipids

Untargeted lipidomics analysis was performed on an Orbitrap IQ-X Tribrid mass spectrometer (Thermo Fisher Scientific, MA, USA) connected to Vanquish Horizon UHPLC (Thermo Fisher Scientific). AcquireX Deep Scan, a data-driven MS/MS acquisition approach, was used to maximize the coverage of lipids as previously described. The lipids were separated as previously described. Full scan MS (*m/z* 250–1500 in positive ion mode and 250–1600 *m/z* in negative ion mode) was acquired at a resolution of 120,000 (at *m/z* 200), while MS/MS scans were acquired at a resolution of 15,000 (at *m/z* 200).

### 2.11. Data Analysis for Lipidomics

Lipids were identified, and peak areas were integrated using LipidSearch 5.1 (Thermo Fisher Scientific) based on precursor *m*/*z* and MS/MS or MS^3^ spectra. Peak areas were normalized to deuterated internal standards added before extraction and to total lipid levels. Statistical comparisons between ALG13-CDG and controls were conducted using Student’s *t*-test.

### 2.12. Plating of cBOs on Multi-Electrode Array (MEA) and Activity, Network, and Axon Recording

Electrophysiological recordings were conducted using the 6-well high-density MEA MaxTwo system (HD-MEA) from MaxWell Biosystems, CH. Wells were prepared following the MaxWell “Brain Organoid Plating Protocol, V2.0,” with minor modifications. Briefly, 6-well MEA plates were incubated in 1% Tergazyme solution for 2 h at room temperature (RT), washed three times with distilled water, sterilized in ethanol for 30 min, and washed three times again with distilled water. Each well received 1 mL of Neurobasal-A (NBA) medium and was incubated for 48 h for pre-conditioning.

After incubation, 50 µL of 0.07% poly(ethyleneimine) (PEI) was added to each well and incubated for 1 h. Wells were then washed three times with distilled water and air-dried for 1 h at RT. Next, 50 µL of 0.04 mg/mL laminin in NBA was added and incubated overnight. Laminin was aspirated, and cortical brain organoids were placed directly on the electrodes without media for 2–5 min. Subsequently, 50 µL of 0.04 mg/mL laminin in NBA was slowly added to each well, and the plates were incubated for 2 h. For each organoid line, four MEA wells were prepared. After the 2 h incubation, 1 mL of NBA was gently added to each well. The following day, half of the media was replaced with fresh NBA.

Organoids were maintained by changing the media every 3–4 days and at least 24 h before MEA recordings on days 120, 124, 133, and 140. Recordings were performed using MaxLab software (https://www.mxwbio.com/products/maxlab-live, accessed on 2 January 2026). The built-in “Activity Scan” protocol was used to identify active areas and assess firing rates. All parameters were kept at default except the recording duration, which was extended to 60 s to better detect slower activity. This was followed by the “Network Assay” protocol to evaluate neuronal network firing, using the neuronal units parameter. Lastly, the “Axon Tracking Assay” was performed using block parameters to detect axonal signal propagation.

For each protocol, wherever feasible, data were averaged across the four wells per sample. Statistical significance was determined using two-way Anova and Šídák post hoc tests.

## 3. Results

### 3.1. Study Cohort

The study included four ALG13-CDG patients carrying the common de novo c.320A>G, N107S variant. Two patients (CDG-0458 and CDG-10175) were previously reported, while two (CDG-11740 and CDG-11816) are newly described. All patients presented with neurological symptoms, including developmental delay, seizures (infantile spasms), intellectual disability, and delayed or absent speech (Table S1). CDG-11740 achieved seizure control on a ketogenic diet, but others did not. Additionally, CDG-1017 and CDG-11816 exhibited central hypotonia, and CDG-11816 and CDG-11740 had ophthalmological abnormalities. CDG-11740 also had hearing loss (Table S1).

### 3.2. X-Inactivation Skewing in ALG13-CDG

Contrary to previous reports suggesting random X-inactivation, three of four patient fibroblast lines showed complete X-inactivation skewing, with CDG-11740 as the exception, exhibiting random inactivation (48% and 52% AR allele methylation; Figure 1B,C). However, cortical organoids derived from all ALG13-CDG iPSCs displayed complete skewing. Control lines confirmed the reliability of this methodology.

### 3.3. Proteomic Analysis of ALG13-CDG hCOs

**Figure 2:**
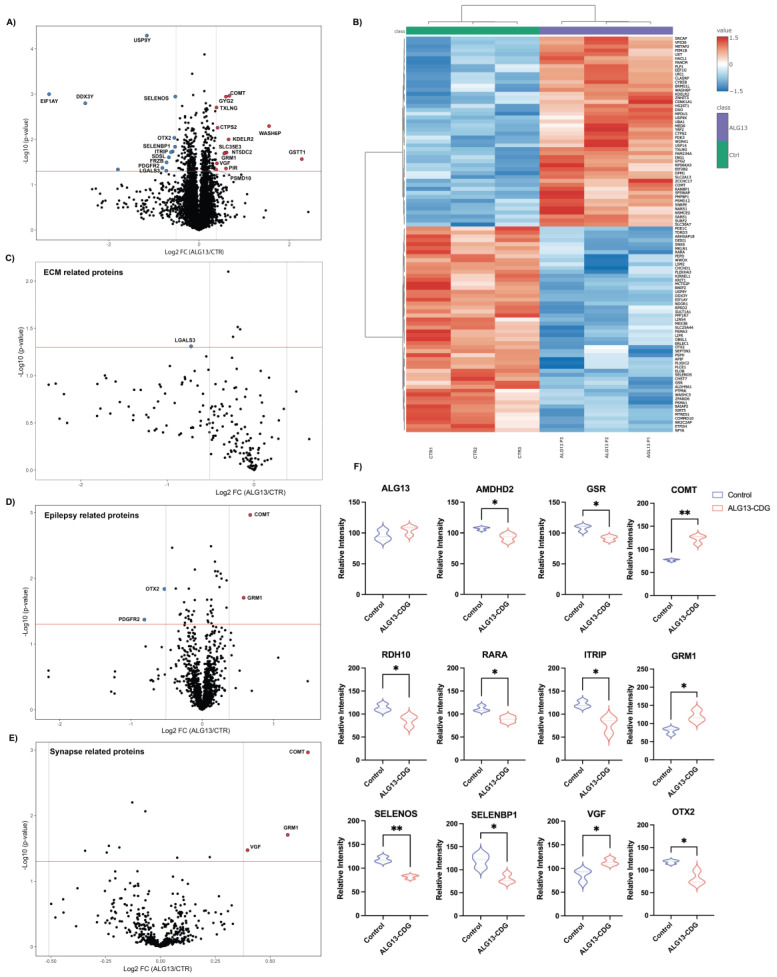
Protein changes in ALG13-deficient hCOs. (**A**) Volcano plot depicting the differentially expressed proteins in ALG13-CDG. The X-axis is log_2_ fold-change (ALG13-CDG/controls), and the Y-axis is the negative logarithm of the *p*-value from a *t* test for significance as indicated. The horizontal dashed red line represents the cutoff for significance (<0.05). Protein names are provided for peptides that have a *p* value < 0.05 and a fold change of 1.3. Left-shifted proteins are downregulated (blue dots), and right-shifted proteins are upregulated (red dots) in ALG13-deficient brain organoids. (**B**) Heatmap of significantly altered proteins with *p* value < 0.05. Volcano plots depicting the differential expression of proteins related to (**C**) extracellular matrix (ECM), (**D**) epilepsy, and (**E**) synapse. (**F**) Violin plots for specific proteins (* *p* value < 0.05; ** *p* value < 0.01).

Global proteomic analysis of three ALG13-CDG and three control cortical brain organoid lines identified 8750 proteins, with 321 significantly altered (Figure 2A,B). Altered proteins included those critical for neuronal function, ER stress, oxidative stress, ECM integrity, and neuronal migration (Figure 2A–F). Specifically, SELENOS, TXLNG, PSMD10, and APIP were dysregulated, impacting ER stress-related apoptosis, while proteins such as OTX2, LGALS3, RDH10, and VGF, essential for brain development, also showed changes. Dysregulation of SELENOS, SELENBP1, and VGF indicated altered lipid metabolism, and downregulation of ITPRIP and PDGFRB indicated calcium ion homeostasis dysregulation.

In examining ECM-associated dysregulation, proteins like LGALS3, HS3ST1, and KRIT1, vital for neuronal development [22,23,24], were downregulated (Figure 2A,B,F). Additionally, RARA and RDH10, important for neuronal migration, were downregulated. Epilepsy-related proteins, including upregulation of GRM1 and COMT and downregulation of PDGFRB and OTX2, were noted (Figure 2D). Synaptic function proteins, such as GRM1, COMT, and VGF, showed upregulation, and AMDHD2, crucial for GlcNAc catabolism [25], was downregulated.

Notably, Y-linked genes, like EIF1AY, appeared downregulated in ALG13-CDG hCOs, consistent with the sex distribution in our cohort and known Y-linked gene downregulation in females [26]. Differences between male and female controls were negligible, suggesting no gender-related bias in our findings.

### 3.4. ALG13-CDG hCOs Exhibit Distinct N-Glycosylation Remodeling

**Figure 3:**
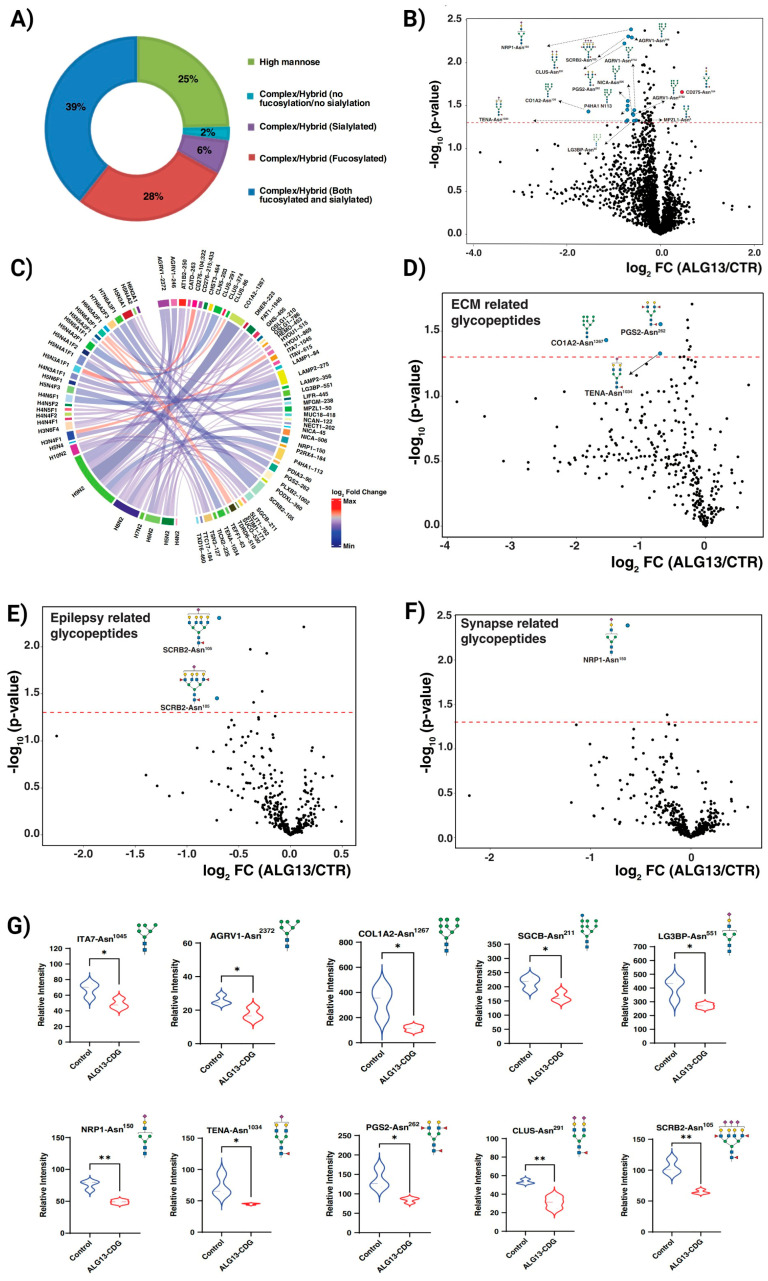
Site-specific glycosylation changes and glycosylation remodeling of different pathways in patient-derived ALG13-CDG hCOs. (**A**) All quantified glycopeptides were categorized based on their glycans, with corresponding percentages provided. (**B**) Volcano plot depicting the differentially expressed glycopeptides in ALG13-CDG. The X-axis is log_2_ fold-change (ALG13-CDG/controls), and the Y-axis is the negative logarithm of the *p*-value from a *t* test for significance as indicated. The horizontal dashed red line represents the cutoff for significance (<0.05). Some of the changing glycopeptides are marked in red circles, and the glycoproteins’ names, glycosylation sites, and glycan structures are drawn. (**C**) Differential chord diagram depicting significantly changing glycopeptides with the proteins and site of attachment in ALG13-CDG as compared to control hCOs. Proteins with different glycosylation sites are indexed on the right of the diagram and connected via chords to the respective identified glycan structures on the left. Asn^x^ represents the asparagine at the amino acid site “x” in the corresponding protein sequence. The fold-change pattern is color-coded. Putative structures are shown using Symbol Nomenclature for Glycans (SNFG). *p* < 0.05 (*), *p* < 0.01 (**). Volcano plots depicting the differential expression of glycopeptides from proteins related to (**D**) extracellular matrix (ECM), (**E**) epilepsy, and (**F**) synapse. The X-axis is log2 fold-change (ALG13-CDG/controls), and the Y-axis is the negative logarithm of the *p*-value from a *t* test for significance as indicated. The horizontal dashed red line represents the cutoff for significance (<0.05). Some of the changing glycopeptides are marked in red circles, and the glycoproteins’ names, glycosylation sites, and glycan structures are drawn. (**G**) Violin plots showing relative intensities of several downregulated glycopeptides in ALG13-CDG derived from numerous proteins associated with these pathways. *p* < 0.05 (*) and *p* < 0.01 (**).

Our N-glycoproteomics analysis of ALG13-CDG hCOs identified 2890 glycopeptides with 282 unique N-glycan structures across 510 sites on 412 glycoproteins. Approximately 25% of these glycans were high mannose, with Man5 and Man8 as the most common types, while the remaining 75% were complex/hybrid (Figure 3A and Figure S5A). Among 66 glycopeptides from 45 glycoproteins showing significant glycosylation changes, 60 glycopeptides exhibited reduced abundance in ALG13-deficient fibroblasts, indicating a broad glycosylation deficit across patients (Figure 3B and Figure S5B). Partial Least Squares Discriminant Analysis (PLS-DA) further confirmed glycopeptide-level distinctions between ALG13-CDG and control hCOs (Figure S5C).

A subset of 23 hypoglycosylated glycopeptides was associated with pathways essential for lysosomal function, lipid metabolism, neuronal migration, apoptosis, and synaptic function [9,27,28,29,30,31,32,33] (Figure 3B). Although not all glycopeptides reached statistical significance, proteins involved in ECM, epilepsy, and synapse-related pathways exhibited reduced glycosylation, potentially contributing to neuronal and synaptic instability (Figure 3D–G). Given that GlcNAc-bisected glycans are predominantly brain-specific, we noted approximately 21% (609 glycopeptides) were GlcNAc-bisected, with a global reduction in ALG13-CDG hCOs, prominently affecting MUC18, PODXL, and TEF1 (Figure S5D).

### 3.5. N-Glycomic Analysis Reveals Distinct Differences in ALG13-CDG hCOs

**Figure 4:**
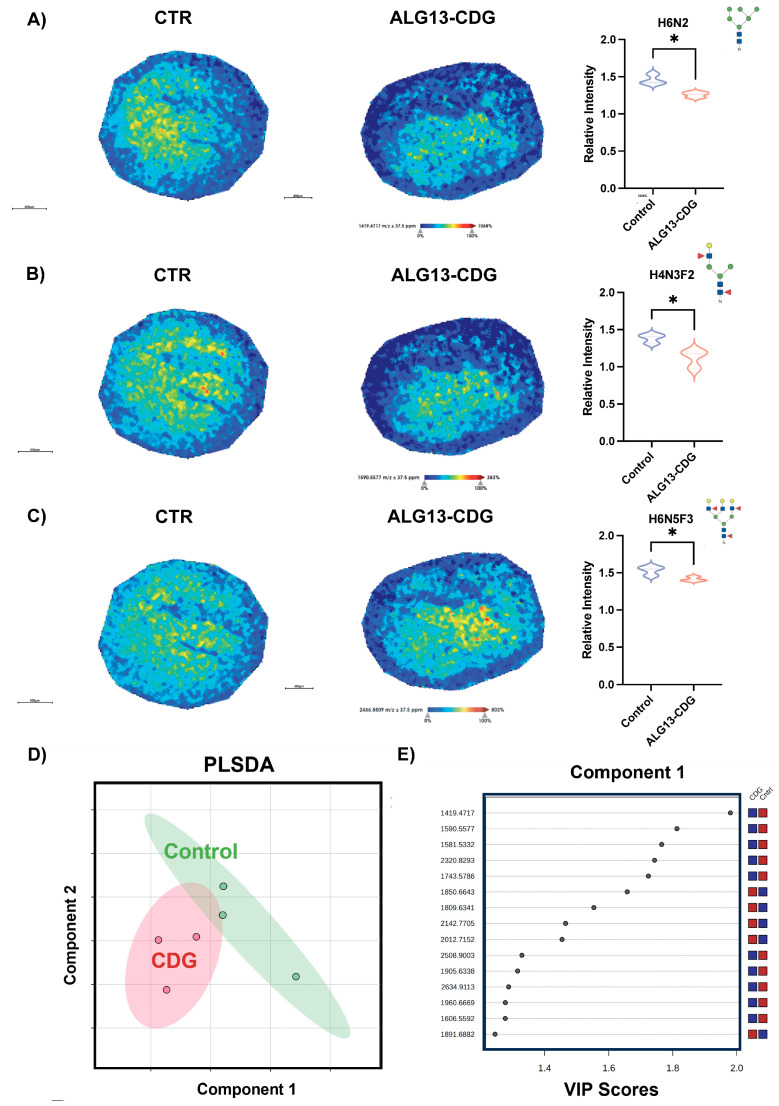
N-glycomic comparison of ALG13-deficient hCOs to controls. MALD-MSI representative images of *m/z* (**A**) 1419.4717 H6N2, (**B**) 1590.5577 H4N3F2, and (**C**) 2466.8809 H6N5F3 N-glycans for control and ALG13-deficient cortical organoids with corresponding relative abundance and N-glycan structure on the right. (**D**) Partial least squares discriminant analysis of the top 15 N-glycans separating the groups with corresponding (**E**) VIP score plots. Shaded areas are 95% confidence intervals. Each point represents 1 patient. Intensity gradient from blue (least abundant) to red (most abundant). Scale bars are below the images. * *p* < 0.05, using Student’s *t*-tests.

Glycan imaging identified 57 N-glycans in both ALG13-deficient and control hCOs (Figure S2A). Further univariate partial least squares discriminant analysis revealed N-glycan species of high mannose, bisecting, and multiantennary N-glycans were prominent discriminators between ALG13-CDG and control (Figure 4A,B). Notably, brain-specific N-glycan species [34] *m/z* 1257.4173 H5N2, *m/z* 1688.6083 H3N5F1, and *m/z* 1996.7276 H4N5F2 were present in both groups without significant differences (Figure S2B–D), supporting the validity of our hCO model (Figure S2B–D). Notable decreases were observed in *m/z* 1419.4717 H6N2, *m/z* 1590.5577 H4N3F2, and *m/z* 2466.8809 H6N5F3 in ALG13-CDG hCOs compared to controls (Figure 4A–C). These differences align with glycoproteomics data indicating altered H6N2 glycosylation in proteins linked to neuronal function, metabolism, and migration.

### 3.6. Cell Type-Specific Transcriptional Alterations Highlight Disrupted Neurodevelopmental Pathways in ALG13-CDG hCOs

**Figure 5:**
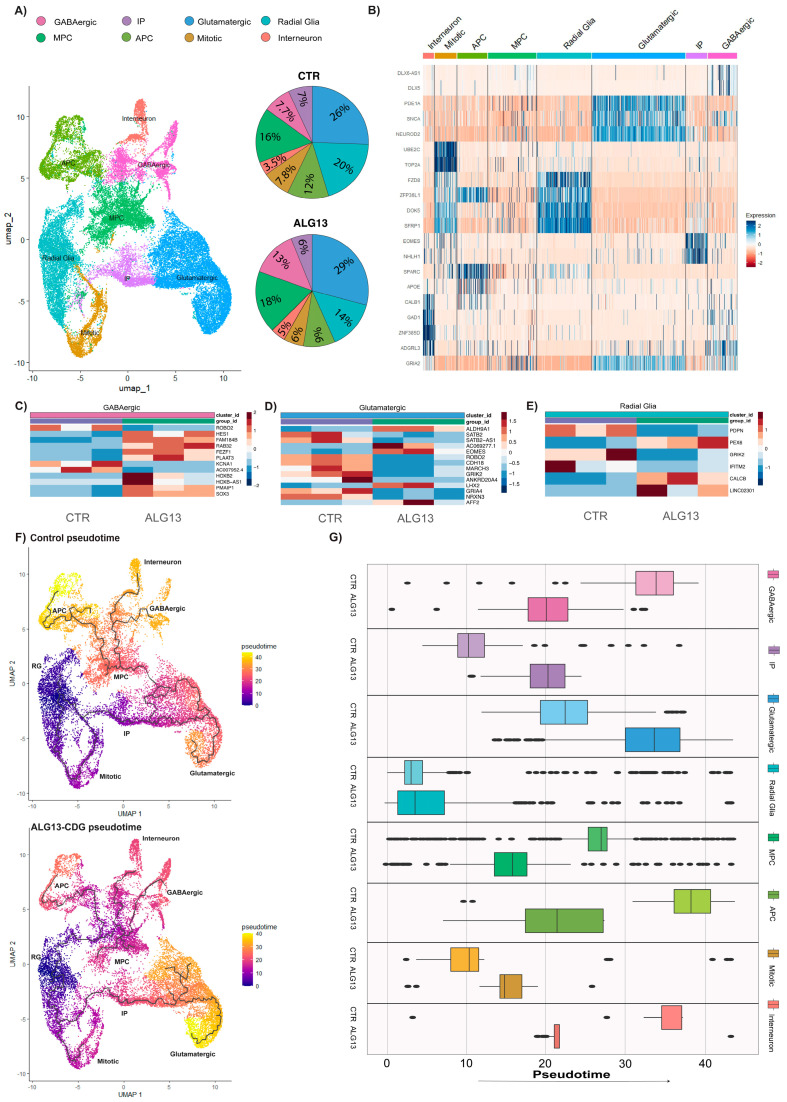
scRNAseq comparison between ALG13-CDG and controls shows an irregular developmental trajectory and alteration in transcripts important for brain development and function. (**A**) UMAP of integrated scRNAseq data that shows the different cell clusters identified in both ALG13-CDG and controls. Percent cell in each cluster for ALG13-CDG and control hCOs. (**B**) Heatmaps of the top markers used to identify the cluster. (**C**–**E**) Heatmaps of differential gene expression analysis for clusters with significant gene changes. (**F**) Trajectory analysis graph for control and ALG13-CDG hCOs, showing the development trajectory of different cell types through color coding associated with pseudotime. (**G**) Boxplots quantifying the pseudotime per cluster in control and ALG13-CDG hCOs.

To assess gene expression differences in ALG13-CDG hCOs, we conducted single-cell RNA sequencing (scRNA-seq) and identified the following eight cell types: radial glia (RG), mitotic radial glia, intermediate progenitors (IP), glutamatergic neurons, GABAergic neurons, interneurons, multipotent progenitor cells (MPCs), and astrocyte progenitor cells (APCs) (Figure 5A and Figure S4). Cluster analysis revealed no significant differences in cell percentages between ALG13-CDG and control hCOs (Figure 5A). However, differential gene expression analysis showed significant dysregulation in RG, glutamatergic, and GABAergic cells, particularly in genes linked to cell migration, apoptosis, axon guidance, oxidative stress, lipid metabolism, and calcium ion regulation (Figure 5C–E).

In neurogenesis, HES1 and SOX3 were downregulated in the GABAergic cluster, and EOMES and LHX in the glutamatergic cluster (Figure 5C,D). PLAAT3 and PEX6, essential for lipid metabolism, were downregulated in the GABAergic and RG clusters, respectively (Figure 5C–E). Additionally, the long non-coding RNA AC007952.4, associated with oxidative stress, was upregulated in ALG13-CDG, alongside altered transcripts linked to neuronal migration (*ROBO2*, *SATB2*, *FEZF1*, and *PDPN*) and upregulation of KCNA1 and glutamate receptors (*GRIK2* and *GRIA4*) (Figure 5C–E).

**Figure 6:**
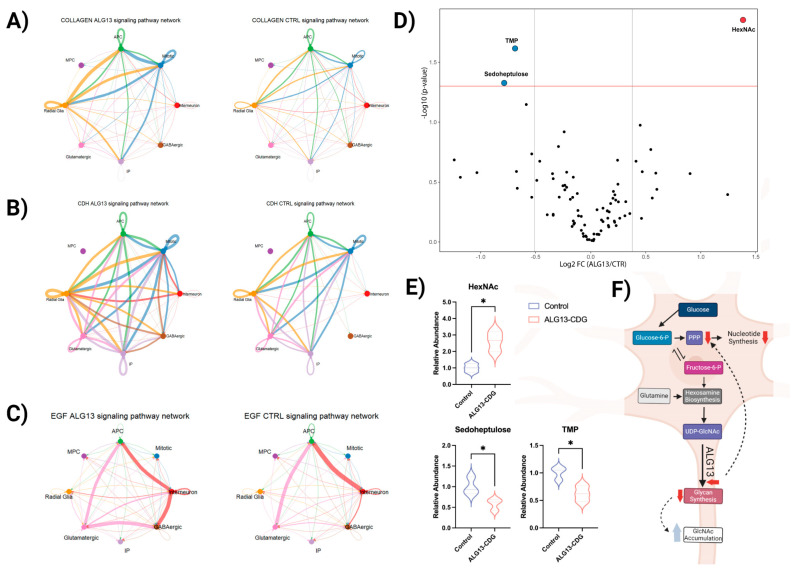
Remodeling of cell communication networks and metabolites in ALG13-deficient hCOs (**A**–**C**). Chord diagrams for specific signaling pathways crucial for extracellular matrix function (**A**), neuronal migration (**B**), and calcium ion homeostasis (**C**). Chords connecting the cell nodes indicate communication between the cell types in regard to the pathways listed. The width of the cord correlates with the weight/amount of communication. (**D**) Volcano plot comparing ALG13-deficient brain metabolites to controls. The X-axis is log2 fold-change (ALG13-CDG/controls), and the Y-axis is the negative logarithm of *p*-value from a *t* test for significance as indicated. The horizontal dashed red line represents the cutoff for significance (<0.05). Metabolite names are provided for metabolites that have *p* value < 0.05 and a fold change of 1.3. (**E**) Violin plots for significantly changing metabolites (* *p* < 0.05). (**F**) Schematic representation of the metabolic alterations in ALG13-deficient hCOs.

CellChat analysis indicated dysregulation in cell communication networks, with heightened activity in collagen, cadherin, and EGF pathways, which may impact ECM function, neuronal migration, and calcium ion homeostasis (Figure S6 and Figure 6A–C). Trajectory analysis showed delayed development of the glutamatergic population in ALG13-CDG hCOs, while MPC, APC, and inhibitory neuron populations developed earlier than in controls (Figure 5F,G).

### 3.7. Metabolic Analysis Reveals Limited Alterations in ALG13-CDG hCOs

Congenital disorders of glycosylation (CDGs) often induce global metabolic rewiring [10,35]. To assess ALG13 deficiency’s impact on metabolism, we performed targeted metabolomics, focusing on GlcNAc-related pathways, including glycolysis, hexosamine biosynthesis, glutamine metabolism, and the pentose phosphate pathway (PPP). Although many metabolites were quantified, ALG13-deficient hCOs showed minimal metabolic differences from control (CTR) hCOs (Figure 6D and Figure S7). Notably, PPP metabolites like TMP and D-Sedoheptulose were downregulated, while HexNAc levels (GlcNAc, GalNAc, and ManNAc) were significantly elevated (Figure 6E).

### 3.8. Lipidomic Profiling Identifies Broad Alterations in Phospholipid Composition in ALG13-CDG hCOs

**Figure 7:**
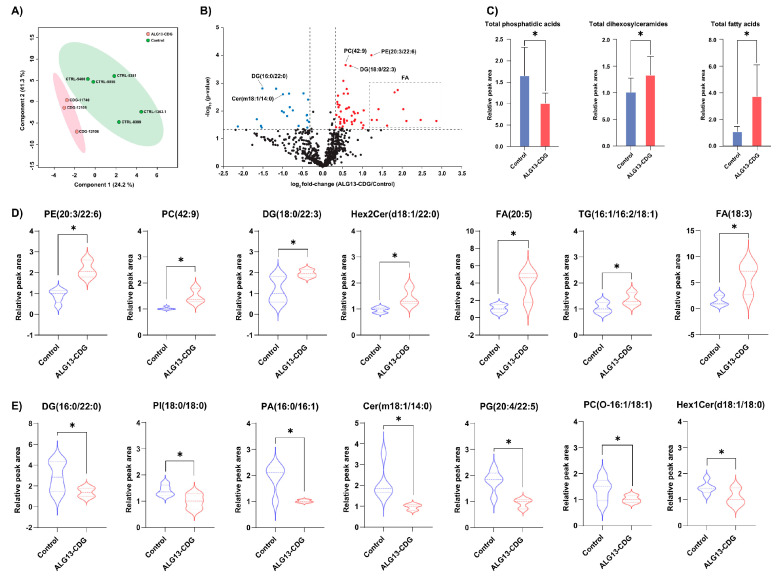
Lipid alterations in ALG13-CDG hCOs. (**A**) Scores plot from partial least squares discriminant analysis of all identified lipids. (**B**) Volcano plot depicting lipidomics changes in ALG13-CDG hCOs. PE (20:3/22:6), PC (42:9), DG (18:0.22:3), and several FA species were significantly increased, while DG (16:0/22:0) and Cer (m18:1/14:0) were significantly decreased. (**C**) Total levels of subclasses of lipids with significant alterations in ALG13-CDG hCOs. (**D**) Violin plots showing lipid species that are significantly increased in ALG13-deficient hCOs. (**E**) Violin plots showing the lipids that are significantly decreased in ALG13-deficient hCOs. * *p* value < 0.05. Abbreviations: PE, phosphatidylethanolamine; PC, phosphatidylcholine; DG, diglyceride; Hex2Cer, dihexosylceramide; FA, fatty acid; TG, triglyceride; PI, phosphatidylinositol; PA, phosphatidic acid; Cer, ceramide; PG, phosphatidylglycerol; Hex1Cer, monohexosylceramide.

Global lipidomic profiling of ALG13-CDG and control hCOs quantified 676 lipids across 23 phospholipid subclasses. Distinct lipid profiles separated ALG13-deficient hCOs from controls (Figure 7A), with significant lipid alterations highlighted in a volcano plot (Figure 7B). In ALG13-CDG hCOs, levels of lipids such as PE (20:3/22:6), PC (42:9), Hex2Cer (d18:1/22:0), and several FA species were increased, while DG (16:0/22:0) and Cer (m18:1/14:0) were decreased. Total PA levels significantly decreased, whereas Hex2Cer and FA increased in ALG13-deficient hCOs (Figure 7C). Overall, 60 lipid species, including PE, Hex2Cer, and FA, showed significant increases (fold change > 1.25, *p* < 0.05) in ALG13-CDG (Figure 7D), while 27 species decreased (fold change < 0.8, *p* < 0.05), as shown in Figure 7E.

### 3.9. ALG13-CDG Cortical Organoids Exhibit Reduced Activity and Impaired Neuronal Network Maturation and Activity

**Figure 8:**
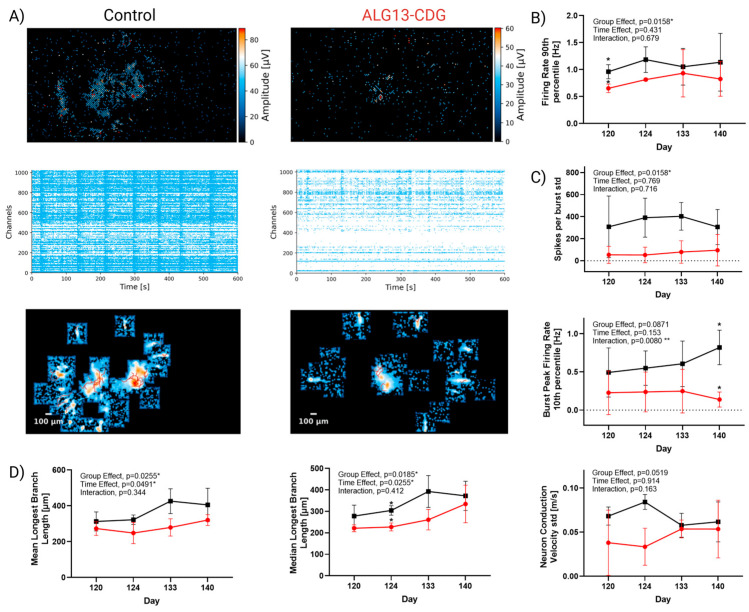
Neuronal network deficits in ALG13-CDG hCOs. (**A**) Representative amplitude maps, raster plots, and axon tracking maps from day 120–140 hCOs for control (left) and ALG13-CDG (right). Line graph summary of MEA data from days 120, 124, 133, and 140. (**B**) Summary for activity metric Firing Rate 90th Percentile [Hz]. (**C**) Summary for network metrics: Spike per Burst, per standard deviation, and burst peak firing rate 10th percentile [Hz]. (**D**) Summary for axon metrics: mean longest branch length [um], median longest branch length [um], and neuron conduction velocity [m/s]. Data were analyzed by two-way ANOVA with Šídák post hoc correction. * *p* < 0.05; ** *p* < 0.01. Error bars indicate SEM.

ALG13-CDG hCOs showed significantly reduced 90th percentile firing rates (Figure 8B, Group Effect *p* = 0.0158), indicating that even the most active neurons in ALG13-CDG fire less than controls and are hypoactive. Spike per burst per standard deviation (Figure 8C, Group Effect *p* = 0.0158) is also diminished in ALG13-CDG, which indicates a more immature network, as the variability in spike numbers within bursts is uniform and weaker. The burst peak firing rate 10th percentile dynamics is also abnormal (group × time interaction, *p* = 0.0080), indicating hypoactivity over time and worsening network coordination.

Axonal signal tracking showed shorter mean and median longest branch lengths (Figure 8D) in ALG13-CDG (Group Effect *p* = 0.0255 and *p* = 0.0185, respectively), indicating neuronal immaturity and deficits in axon extension. A trend toward decreased conduction velocity variance (Group Effect *p* = 0.0519) was also seen in ALG13-CDG (Figure 8D), which is consistent with immature neurite outgrowth and signal propagation.

## 4. Discussion

This study presents the first biochemical evidence of brain-specific N-glycosylation abnormalities in ALG13-CDG, addressing a longstanding gap in understanding the molecular basis of this disorder’s neurological symptoms. While individuals with ALG13-CDG often have normal serum glycosylation profiles, our findings reveal significant glycosylation defects in brain tissue, aligning with the predominantly neurological phenotype. These observations have important diagnostic implications, indicating that peripheral glycosylation assays may underestimate disease burden and that brain-relevant model systems are required to capture disease-relevant molecular pathology. Through multiomic analysis, we have identified key pathways potentially contributing to seizures, intellectual disability, and developmental delays in ALG13-CDG, providing a mechanistic framework to inform future therapeutic development.

### 4.1. X-Inactivation Skewing in ALG13-CDG

Our study identified significant X-inactivation skewing in three of four ALG13-CDG fibroblast lines, contrasting with previous reports of random X-inactivation in these patients [36]. Extensive X-inactivation skewing was also observed in iPSC-derived cortical organoids from ALG13-CDG patients, highlighting the potential role of X-linked epigenetic regulation in disease pathology. Although XCI skewing could theoretically contribute to phenotypic variability, the limited cohort size precludes robust association analyses. Importantly, highly consistent molecular, cellular, and electrophysiological phenotypes were observed across patient-derived organoids despite variable XCI patterns, suggesting that XCI bias is unlikely to be a major determinant of the core pathological features identified here. Future studies incorporating larger cohorts, isogenic controls, and allele-specific analyses will be required to more definitively assess the potential contribution of XCI dynamics to clinical heterogeneity in ALG13-CDG.

### 4.2. ECM Dysregulation Contributes to Neurodevelopmental Defects in ALG13-CDG

Our results demonstrate that hypoglycosylation of key ECM components, including collagen (CO1A2) and proteoglycan (PGS2, Figure 3), could disrupt ECM integrity, an essential factor for neuronal migration and axon extension during cortical development. Neuronal migration depends on interactions between ECM proteins and radial glial cells, which help direct neurons to their proper locations [29]. Altered glycosylation of integrins (ITA7, LG3BP) and the upregulation of PDPN may further destabilize these critical cell-ECM interactions [30], potentially explaining the developmental delays [28] and seizure phenotypes [37] observed in ALG13-CDG.

From a translational perspective, these findings suggest that ECM-associated signaling pathways may represent underexplored therapeutic targets in ALG13-CDG and related CDGs. Reduced levels of ECM-related proteins, such as RDH10 and RARA (Figure 2B,F), linked to microcephaly, indicate that ECM dysregulation may contribute to the structural brain abnormalities reported in ALG13-CDG [38]. Furthermore, as the ECM is crucial for axon extension, ECM disruption may also underlie the reduced axonal extension in ALG13-CDG (Figure 8A,D).

### 4.3. ER Stress-Driven Oxidative Dysregulation in ALG13-CDG

Multiple ER stress-associated genes, including TXLNG, PSMD10, and SELENOS (Figure 2A), were dysregulated in ALG13-deficient cortical organoids, consistent with impaired protein folding and activation of the unfolded protein response. Chronic ER stress is likely to exacerbate oxidative imbalance through increased reactive oxygen species generation, a mechanism particularly deleterious in the metabolically vulnerable developing brain [39,40,41]. Single-cell transcriptomic analyses revealed upregulation of oxidative stress markers within GABAergic neuronal populations (Figure 5C), indicating cell type-specific susceptibility. These observations suggest that oxidative stress pathways may act as important modifiers of disease severity and represent potential targets for adjunctive therapeutic strategies aimed at enhancing neuronal resilience rather than directly correcting glycosylation defects.

### 4.4. Calcium-Signaling Abnormalities Contribute to Neurological Pathology in ALG13-CDG

Our analysis identified hypoglycosylation of SGCB and AGRV1, proteins important for maintaining calcium homeostasis [31,32], in ALG13-CDG. Disrupted calcium regulation has significant implications for neuron function, as calcium signaling is crucial for cognitive processes [42], synaptic plasticity, and neuronal excitability. Further supporting this, our proteomics data revealed downregulation of ITRRIP and PDGFRB (Figure 2A), both of which play roles in calcium signaling and have been associated with epilepsy [42]. The disruption of calcium signaling may, therefore, contribute to the complex neurological symptoms in ALG13-CDG, including seizures, developmental delays, and intellectual disability. Furthermore, altered EGF signaling between glutamatergic and GABAergic clusters (Figure 6C) in ALG13-CDG suggests a broader impact on calcium-dependent developmental pathways [43], potentially affecting excitatory and inhibitory neuron development (Figure 5C–G). Collectively, these findings indicate that glycosylation-dependent modulation of calcium signaling pathways contributes to the neurological manifestations of ALG13-CDG and reinforces the concept that disease pathogenesis arises from coordinated disruption of multiple signaling systems rather than a single linear defect.

### 4.5. Lipid and Metabolic Disruption Aligns with Proteomic and Transcriptomic Defects in ALG13-CDG

Our lipidomic profiling identified significant disruptions in lipid metabolism in ALG13-CDG. Notable increases in lipid species (Figure 7B–D), including FA(22:1), FA(22:4), and FA(22:6), suggest peroxisomal dysfunction [44], supported by decreased PEX6 transcript levels (Figure 5C). Elevated lactosylceramide (Hex2Cer) levels, linked to oxidative stress and mitochondrial dysfunction [45], may contribute to the seizure and developmental delay phenotypes observed in ALG13-CDG. Decreased levels of PA, essential for dendritic spine maturation and synaptic plasticity [46], further suggest a connection between lipid metabolism dysregulation and the intellectual disability phenotype.

Elevated HexNAc levels (Figure 6E) are consistent with impaired utilization of UDP-GlcNAc resulting from ALG13 dysfunction, providing a link between defective glycosylation and altered metabolic homeostasis. Downregulation of AMDHD2 (Figure 2F), a key enzyme in GlcNAc catabolism, supports this interpretation. While decreased pentose phosphate pathway (PPP) metabolites suggest altered metabolic flux and redox imbalance, their causal contribution to the observed phenotypes cannot be determined from the present data and will require targeted metabolic flux and rescue studies in future work.

### 4.6. Network-Level Vulnerability and Excitatory/Inhibitory Imbalance in ALG13-CDG

Neuronal hyperexcitability is a known driver of seizures [47], and our findings reveal hypoglycosylation of AT1B2 (Figure 3), a glycosylated component of the Na+/K+ pump, in ALG13-CDG. This hypoglycosylation may impair the pump’s ability to regulate membrane potential, increasing the likelihood of hyperexcitability. Additionally, our proteomics and scRNAseq analyses show upregulation of excitatory receptors such as *GRM1*, *GRIK2*, and *GRIA4*, further supporting a hyperexcitable neuronal environment. Although direct cell-level functional assays would further strengthen causal inference, the study was designed to interrogate network-level consequences of ALG13 deficiency. The convergence of transcriptomic alterations affecting excitatory and inhibitory neuronal programs with highly reproducible electrophysiological abnormalities provides strong indirect evidence for disrupted E/I balance.

### 4.7. Paradoxical Hypoactivity Reveals Latent Hyperexcitability and Disrupted E/I Maturation in ALG13-CDG

Despite the molecular evidence of neuronal hyperexcitability, our electrophysiological recordings revealed network hypoactivity in ALG13-CDG hCOs, characterized by reduced firing rates, immature bursting patterns, and shorter neurite extensions. This paradox, where hypoactive networks are present in a seizure-prone disease, suggests the presence of latent hyperexcitability, a state in which neurons are biochemically primed to overactivate despite a subdued baseline functional state. Upregulation of excitatory receptors (e.g., GRM1, GRIA1) may represent an early response to suppressed activity (homeostatic plasticity), increasing the system’s vulnerability to overstimulation or destabilization.

Importantly, latent hyperexcitability has been reported in other neurodevelopmental and seizure disorders, where stress can trigger a transition from hypoactivity to seizure [48,49,50]. This may explain why ALG13-CDG patients develop seizures despite early developmental suppression of neural activity. Furthermore, our pseudotime trajectory data revealed temporal mistiming in neuronal lineage development, with early inhibitory and delayed excitatory neuronal development. Such a mistimed circuit assembly may represent a critical developmental vulnerability window during which therapeutic strategies aimed at stabilizing network maturation could be most effective.

## 5. Conclusions

Together, these findings establish ALG13-CDG as a disorder of brain-specific hypoglycosylation that destabilizes core neurodevelopmental pathways—including ECM organization, ER stress responses, calcium signaling, and metabolic homeostasis—ultimately converging on cortical network dysfunction. By reframing ALG13-CDG as a disorder of network instability rather than an isolated glycosylation defect, this work provides a conceptual framework with direct diagnostic and therapeutic relevance. While future studies must examine additional ALG13 variants and larger cohorts, the consistency of phenotypes across patient-derived organoids supports the robustness of the mechanisms identified here. More broadly, the convergence of multi-pathway dysregulation onto excitatory/inhibitory imbalance suggests shared vulnerabilities across neurologically involved CDGs and highlights network stabilization as a potential therapeutic objective.

## Data Availability

The mass spectrometry proteomics data have been deposited to the ProteomeXchange Consortium via the PRIDE partner repository with the dataset identifier PXD051647. Deidentified metabolomics data have been deposited at the National Metabolomics Data Repository Metabolomics workbench, and its study ID is ST003121. The scRNAseq data have been deposited to Gene Expression Omnibus (GEO) with accession number GSE266155.

## Supplementary Materials

The following supporting information can be downloaded at: https://www.mdpi.com/article/10.3390/cells15020147/s1, Table S1: Clinical Data on the ALG13-CDG Patients in this Study; Figure S1: Pluripotency and Germ Layer Markers for ALG13-CDG iPSCs. All images are at 20X resolution. Nanog, TRA-1-60, Oct4, & SSEA were used as pluripotency markers. SOX17 and FoxA2 staining were used as endoderm markers. CD31 and NCAM were used as mesoderm markers, while Nestin and Pax6 were used as ectoderm markers; Figure S2: N-glycomic comparison of ALG-13-deficient cortical organoids to controls. (A) Unbiased clustering heatmap analysis of all 57 N-glycan features stratifying the groups. MALD-MSI representative images of *m/z*. (B) 1257.4173 H5N2, (B) 1688.6083 H3N5F1, and (C) 1996.7276 H4N5F2 N-glycans for control and ALG13-deficient cortical organoids with corresponding relative abundance and N-glycan structure on the right. Intensity gradients from blue (least abundant) to red (most abundant). Scale bars are below the images; Figure S3: Bulk differential gene expression analysis of scRNAseq data reveals alteration in genes important for neuronal excitation, lipid metabolism, extracellular matrix, and lipid metabolism; Figure S4: Dotplot of transcripts associated with specific cell populations. Percent expressed is represented by the size of the dot, and expression level is represented on a scale from −1 (blue) to 2 (red); Figure S5: Site-specific glycosylation changes in patient-derived ALG13-CDG hCOs. (A) High mannose glycans containing glycopeptides are shown with numbers representing glycopeptides found with increasing hexose units. (B) Heatmap of significantly changing glycopeptides (*p*-value < 0.05) with different high-mannose and complex/hybrid glycan moieties. The pattern is color-coded. (C) Partial Least Squares Discriminant Analysis (PLS-DA) based on reporter ion intensities for all identified glycopeptides of ALG13-CDG patients and controls. The percentage of total variance associated with each component is shown in brackets with the axis label. (D) Volcano plots depicting the downregulation of glycopeptides carrying the bisected glycan composition in ALG13-CDG. Putative structures are shown using Symbol Nomenclature for Glycans (SNFG); Figure S6: Ranked flow chart showing the relative information flow from signaling pathways identified in hCOs. The cyan color indicates flow from controls, and the punch-pink color indicates flow from ALG13-CDG hCOs; Figure S7: Heatmap of metabolites detected in ALG13-deficient and control hCOs. The pattern is color-coded.

## Abbreviations

ALG13, asparagine-linked glycosylation 13; ALG13-CDG, ALG13–congenital disorder of glycosylation; APC, astrocyte progenitor cell; AR, androgen receptor; CDG, congenital disorder of glycosylation; CNS, central nervous system; ECM, extracellular matrix; EGF, epidermal growth factor; E/I, excitatory/inhibitory; ER, endoplasmic reticulum; FA, fatty acid; GABA, gamma-aminobutyric acid; GlcNAc, N-acetylglucosamine; hCO, human cortical organoid; HexNAc, N-acetylhexosamine; iPSC, induced pluripotent stem cell; MPC, multipotent progenitor cell; N-glycan, asparagine-linked glycan; PA, phosphatidic acid; PC, phosphatidylcholine; PE, phosphatidylethanolamine; PPP, pentose phosphate pathway; RG, radial glia; scRNA-seq, single-cell RNA sequencing; ACTH, adrenocorticotropic hormone.
